# Separation and Identification of 1,2,4-Trihydroxynaphthalene-1-*O*-glucoside in *Impatiens glandulifera* Royle

**DOI:** 10.3390/molecules18078429

**Published:** 2013-07-17

**Authors:** Jan Tříska, Naděžda Vrchotová, Jan Sýkora, Martin Moos

**Affiliations:** 1 Laboratory of Metabolomics and Isotopic Analyses, Global Change Research Centre, Academy of Sciences of the Czech Republic, Branišovská 31, České Budějovice 370 05, Czech Republic; E-Mails: vrchotova.n@czechglobe.cz (N.V.); moos.m@czechglobe.cz (M.M.); 2 Department of Organic Synthesis and Analytical Chemistry, Institute of Chemical Process Fundamentals, Academy of Sciences of the Czech Republic, Rozvojová 135, Praha 165 05, Czech Republic; E-Mail: Sykora@icpf.cas.cz

**Keywords:** 1,2,4-trihydroxynaphthalene-1-*O*-glucoside, *Impatients glandulifera* Royle

## Abstract

Methanolic extract from lyophilized roots of *Impatiens glandulifera* Royle was analyzed by high performance liquid chromatography using DAD and FLD detection and this revealed one dominant highly fluorescent very unstable substance. The stability of this derivative is strongly dependent on the plant material drying procedure and extraction procedure used. The structure of the substance was established as 1,2,4-trihydroxynaphthalene-1-*O*-glucoside (THNG) according LC-MS and NMR measurements. When lyophilized plant material was extracted with methanol an almost four times higher amount of THNG was found in the extract, compared to the amount of 2-hydroxy-1,4-naphthoquinone obtained, while in the case of the same lyophilized plant material extracted with water there was no THNG in the extract. The main compounds in this case was 2-hydroxy-1,4-naphthoquinone. In the plant material dried at the laboratory temperature and extracted by methanol there are only traces of THNG.

## 1. Introduction

Three species of *Impatiens* grow in Europe: *Impatiens noli-tangere* L., *Impatiens glandulifera* Royle and *Impatiens parviflora* DC. *Impatiens noli-tangere* L. is the original plant species, while *Impatiens glandulifera* Royle and *Impatiens parviflora* DC. are among the invasive plants originally native to Asia that are rapidly spreading across the Europe. The abovementioned *Impatiens* species also contain naphthoquinones in addition to phenolic compounds of the flavonol and caffeic acid type derivatives [1,2]. Naphthoquinones are contained predominantly in the roots and consist mainly of 2-hydroxy-1,4-naphthoquinone (lawsone) and 2-methoxy-1,4-naphthoquinone (lawsone methyl ether). Lawsone from *Lawsonia inermis* L. is still object of the research due to its various usages [3]. The first attempt to describe a trihydroxynaphthalene-glucoside in *Impatiens* species without location of glucose position was done by [4]. There are more information about trihydroxynaphthalene-*O*-glucosides in other plant species which contain the naphthoquinones, for examples: 1,2,4-trihydroxynaphthalene-1,4-di-β-D-glucopyranoside in *Lawsonia inermis* L. [5], THNG was found for the first time in *n*-butanolic fraction of *Lawsonia inermis* L. [6], 7-methyl-1,4,5-trihydroxynaphthalene-4-*O*-glucoside in *Drosera spathulata* Labill. [7], 1,4,5-trihydroxynaphthalene-glucoside in *Carya illinoensis* (Wangenh.) K. Koch [8], several trihydroxynaphthalene glucosides were described in *Juglans mandshurica* Maxim. [9]. 

2-Hydroxy-1,4-naphthoquinone and 5-hydroxy-1,4-naphthoquinone were glucosidized and their acetylated glucosides were tested for their immunosuppressive and immunostimulative properties. It was found that their activity was comparable with clinically used preparation [10]. Naphthoquinones as aglycones have a wide range of biological properties, they are known for their phytotoxicity, extracts from *Impatiens noli-tangere* L., *Impatiens glandulifera* Royle and *Impatiens parviflora* DC. strongly inhibit the germination and growth [11]. Naphthoquinones from *Drosera rotundifolia* L. are used in respiratory difficulties [12]. In experiments on tobacco BY-2 cells naphthoquinones play a role in the cell death [13]. 2-Methoxy-1,4-naphthoquinone has strong *in vitro* anticancer activity against HepG2 cells, experiments that were performed with extracts from *Impatients balsamina* L. [14].

Due to long-term investigation of the biologically active substances in *Impatients* we found that the extract composition varies depending on the type of processing the plant material is subjected to. First, the plant material (roots) was air dried at the room temperature, which corresponds to the recent literature data about drying [6], where in the roots the dominant compound was 2-hydroxy-1,4-naphthoquinone. THNG was not found in this case. In the subsequent lyophilization one dominant peak of a yet unknown substance appeared. Considering these facts, the main objective of our work was to study the conditions of plant material processing with respect to the maximum yield of this unknown substance.

## 2. Results and Discussion

**Figure 1:**
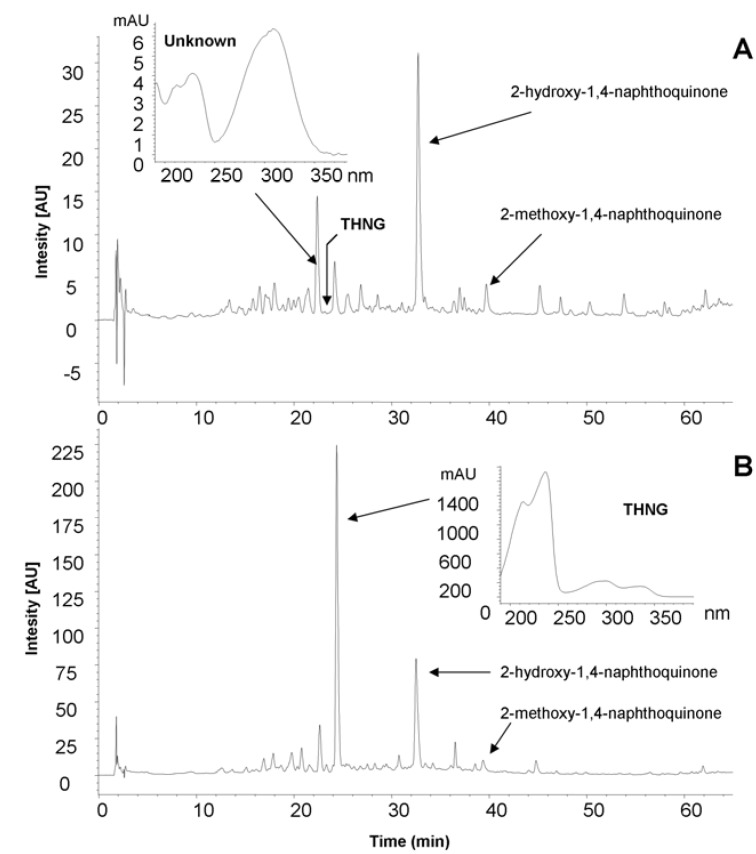
The chromatographic profile (HPLC) of methanolic extracts prepared from the roots of *Impatiens glandulifera* Royle dried at room temperature (**A**) and lyophilized roots (**B**).

**Figure 2:**
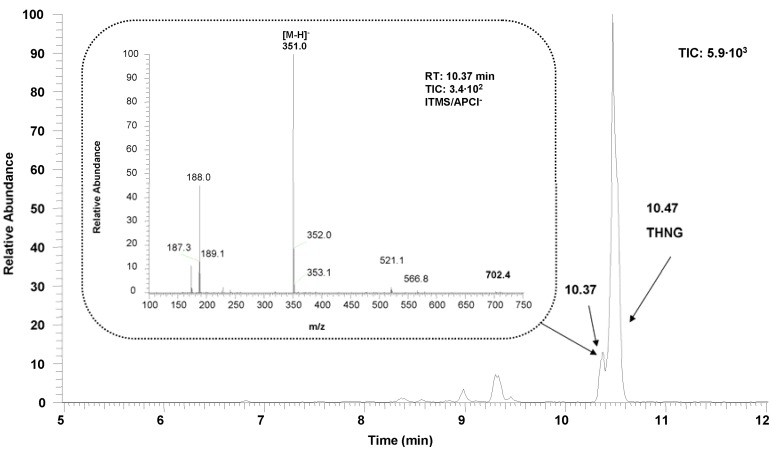
Chromatographic profile of the *Impatiens glandulifera* Royle methanolic extract of the roots measured as total ion current (TIC) by LC-MS (APCI). MS spectrum of THNG is shown on the Figure 3.

For impatiens, we found that chromatographic profile of the extracts varies greatly depending on the method of plant material processing and its time of collection (Figure 1 and Figure 2).

**Figure 3:**
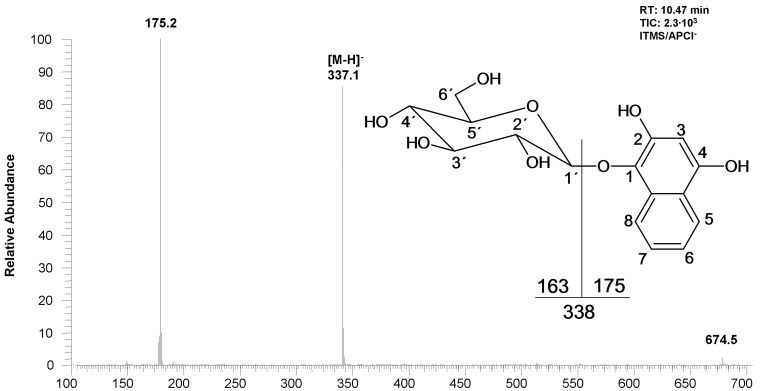
Mass spectrum and chemical structure of the 1,2,4-trihydroxynaphthalene-1-*O*-glucoside.

In the methanolic extracts from lyophilized roots of *Impatiens glandulifera* Royle we detected by liquid chromatography a dominant substance whose UV spectrum is different from that of the very common 2-hydroxy-1,4-naphthoquinone (the chromatographic profiles of the extracts are given in Figure 1 and Figure 2) and which has a high fluorescence. The mass spectrum of the dominant substance was measured by LC-MS APCI in the negative mode (Figure 3). The molecular ion is *m/z* 337^+^ (APCI negative mode) and the most significant fragment in the mass spectra (APCI negative mode) is *m/z* 175^+^ [M−162]^+^. A loss of 162 amu corresponds to anhydroglucose and it is typical for the fragmentation of glucosides. Therefore the compound with the retention time 10.47 min (see Figure 2 and Figure 3) was determined as trihydroxynaphthalene glucoside. 

**Figure 4:**
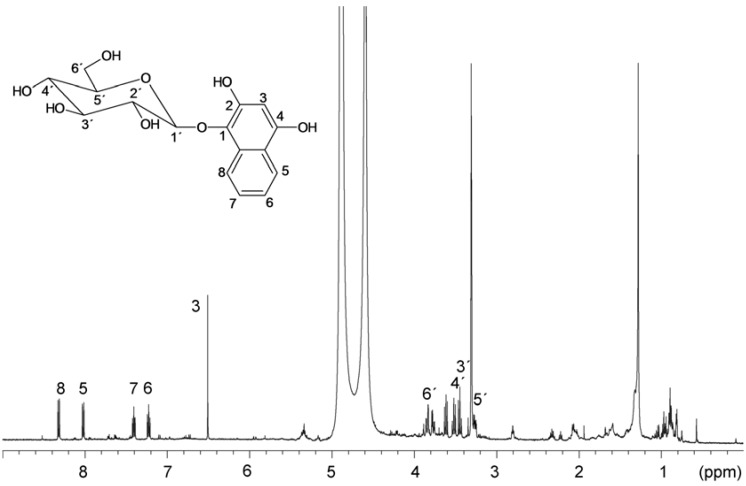
^1^H-NMR spectrum and the signal assignment of 1,2,4-trihydroxynaphthalene-1-*O*-glucoside.

**Figure 5:**
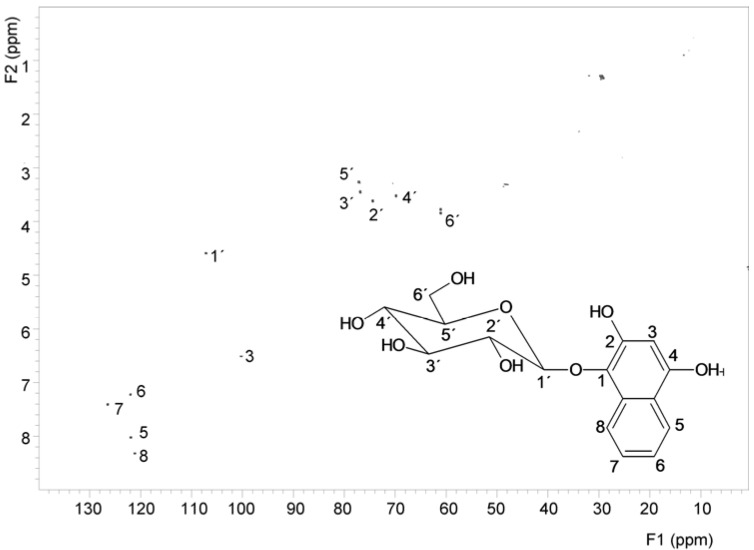
HSQC spectrum and the signal assignment of 1,2,4-trihydroxynaphthalene-1-*O*-glucoside.

Identification of glucose, location of hydroxy groups and binding site of glucose was determined by NMR. The structure of the aglycone was also verified by NMR (Figure 4). Using NMR, it was established that the hydroxy groups are in position 1,2,4 and glucose is linked at position 1 (*i.e*., the structure is 1,2,4-trihydroxynaphthalene-1-*O*-glucoside, THNG). The structure elucidation was based on 2D experiments (COSY, HSQC and HMBC), given in Figure 5. Particularly this spectrum documents the concentration of the compound in the sample and its purity.

According to the literature THNG was recently found in an *n*-butanolic fraction of *Lawsonia inermis* L. [6]. It is interesting that in the literature relating to the compounds found in *Impatiens* species there is no mention of 1,2,4-trihydroxynaphthalene-1-*O*-glucoside (THNG) [4]. We believe that this fact is related to its very low stability. It is our opinion that the drying procedure and extraction method play a very important role in this fact.

**Scheme 1:**
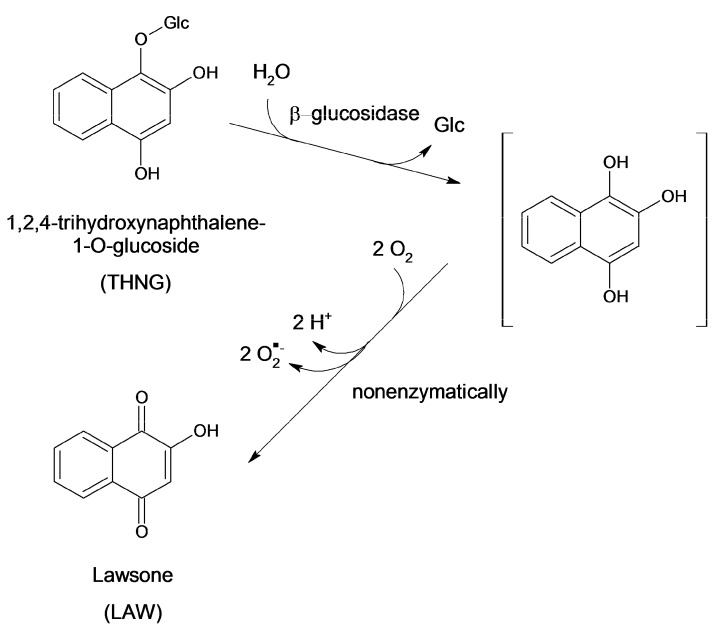
Transformation of 1,2,4-trihydroxynaphthalene-1-*O*-glucoside to 2-hydroxy-1,4-naphthoquinone (lawsone).

THNG can be determined in the methanolic extract of freeze-dried material even after several years, while in the material dried at room temperature there were practically only traces of THNG (Table 1). The solvent used for the extraction also affects the content of THNG. Extraction with water is more suitable for obtaining of 2-hydroxy-1,4-naphthoquinone and 2-methoxy-1,4-naphthoquinone, but THNG is practically not present in the aqueous fraction, although it is more polar than the naphthoquinones (Table 2). THNG was not detected even after subsequent extraction of the material with methanol. The results revealed that the originally present THNG is hydrolyzed to 1,2,4-trihydroxynaphthalene (THN) by *β*-glucosidases, finally yielding 2-hydroxy-1,4-naphthoquinone (lawsone) according to Scheme 1. The intermediate aglycone THN is rather unstable, e.g., after mild acidic hydrolysis of THNG we did not find any trace of THN. The instability of THN is also supported by the literature [15]. The low stability of the aqueous extracts of 7-methyl-1,4,5-trihydroxynaphthalene-4-*O*-glucoside (from *Drosera*) also points this out [7].

**Table 1:** The content of 1,2,4-trihydroxy-naphthalene-1-*O*-glucoside (THNG), 2-hydroxy-1,4-naphthoquinone (**A**) and 2-methoxy-1,4-naphthoquinone (**B**) in *Impatiens glandulifera* Royle (mg/kg).

Year of collection	Part of plant	Drying	Storage	Solvents	THNG	A	B
2008	root	RT	RT	MeOH	ND	48	8
2008	root	−18 °C+LY	RT	MeOH	204	92	13
2008	root	−18 °C+LY	4 °C	MeOH	210	115	7
2011	stem	40 °C	RT	MeOH	2	53	19
2011	stem	−18 °C+LY	RT	MeOH	148	74	11
2011	stem	−18 °C+LY	4 °C	MeOH	176	70	10
2011	stem	−18 °C+LY	−18 °C	MeOH	200	118	15
2011	root	−18 °C+LY	−18 °C	MeOH	826	143	51
2011	leaf	−18 °C+LY	−18 °C	MeOH	247	ND	69
2013	leaf	N_2_(l)+LY	WS	MeOH	230	ND	111
2013	stem	N_2_(l)+LY	WS	MeOH	202	148	20
2013	root	N_2_(l)+LY	WS	MeOH	1359	115	29
2013	leaf	N_2_(l)+LY	WS	H_2_O	ND	499	1301
2013	stem	N_2_(l)+LY	WS	H_2_O	ND	356	58
2013	root	N_2_(l)+LY	WS	H_2_O	98	2277	81

RT: room temperature; −18 °C+LY: frozen in a freezer at −18 °C + lyophilization; N_2_(l) + LY: frozen in liquid nitrogen and lyophilization; WS: without storage; ND: below detection limit.

**Table 2:** The content of 1,2,4-trihydroxynaphthalene-1-*O*-glucoside (THNG), 2-hydroxy-1,4-naphthoquinone (**A**) and 2-methoxy-1,4-naphthoquinone (**B**) in *Impatiens glandulifera* Royle (mg/kg) after subsequent extraction (water followed by methanol).

				THNG		A		B	
Year of collection	Part of plant	Drying	Storage	H_2_O	MeOH	H_2_O	MeOH	H_2_O	MeOH
2008	root	RT	RT	ND	ND	46	16	4	1
2008	root	LY	RT	ND	ND	740	335	17	23
2008	root	LY	4 °C	ND	ND	1121	484	15	20
2011	stem	40 °C	RT	ND	ND	128	47	ND	ND
2011	stem	LY	RT	ND	ND	321	235	43	25
2011	stem	LY	4 °C	ND	ND	263	177	48	24
2011	stem	LY	−18 °C	ND	ND	319	191	54	24

RT: room temperature; LY: lyophilization; ND: below detection limit.

**Figure 6:**
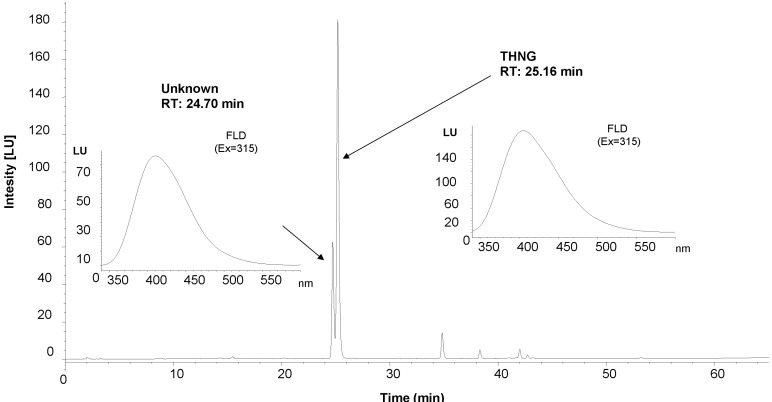
HPLC chromatographic profile of the fresh flower methanolic extract of *Impatiens glandulifera* Royle with FLD detection.

We found that THNG is contained not only in the roots, but in lesser amounts also in aboveground parts of *Impatiens glandulifera* Royle. Figure 6 shows chromatogram of the methanolic extract of fresh flowers and fluorescence spectra of THNG and unknown compound. 

The spectra are very similar, there is only shift in the maximum (+3 nm for the unknown compound compare to THNG). In addition, we have found besides THNG in the lyophilized or fresh materials of *Impatiens* other substances that have similar UV and FLD spectrum as THNG; the mass spectrum this compound is shown in Figure 2. The compound was found in *Impatients parviflora* DC. and *Impatients balsamina* L., not only in the roots, but also in the above-ground parts of the plants. In the roots of *Impatiens glandulifera* Royle we also identified scopoletin, which has been previously described in root cultures of *Impatiens balsamina* L. [16].

## 3. Experimental

### 3.1. Standards

Both 2-hydroxy-1,4-naphthoquinone and 2-methoxy-1,4-naphthoquinone were purchased from the Extrasynthese Lyon Nord, Genay Cedex, France.

### 3.2. Sample Preparation

*Impatiens glandulifera* Royle and *Impatiens parviflora* DC. plants were collected around Ceske Budejovice. A large number of plants (at least 5) has always been sampled at one location to ensure homogeneity of the material. *Impatiens balsamina* L. was purchased in a local shop. It was a pink variety K11. The material was divided into roots, stems and leaves, and cut into smaller pieces of about 10 cm. The plant material was dried under various conditions: at room temperature, at 40 °C and it was also lyophilized (plant material was frozen at −18 °C before lyophilization). After drying, the material was finely shredded using knife grinder, mixed thoroughly and kept at different temperatures (−18 °C, +4 °C and laboratory temperature). The extracts from materials collected in years 2008 and 2011 were prepared in 2011. The flowers were immediately immersed in methanol after being picked at the sampling site. In 2013, the plant material was immediately immersed into liquid nitrogen directly at the sampling site and after transportation to the laboratory and after removal from liquid nitrogen the samples were immediately placed in a freeze drier. After lyophilization the samples were immediately grinded up and extracted.

Plant materials were extracted by several ways:

1) Extraction with 100% methanol for 30 min at the room temperature under continuous shaking, after centrifugation the sediment was washed further twice with methanol and the supernatants were combined.
2) Extraction with distilled water under the same conditions as with methanol and after centrifugation the sediment was washed twice with water, supernatants were combined, then the sediment was extracted with methanol for 30 min at the room temperature under continuous shaking, the sediment after centrifugation was washed twice with methanol and methanolic extracts were combined (Table 2).
3) For NMR measurements the lyophilized roots were not extracted with methanol, but with ethyl acetate. Ethyl acetate fraction was then evaporated in a vacuum evaporator to dryness and the sample was dissolved in small volume of methanol. Extraction with ethyl acetate is not as effective as extraction with methanol, but the resulting extract is relatively clean, containing only a minor impurities. During the extraction with ethyl acetate very polar compounds and free sugars do not pass into extract. After evaporation of the ethyl acetate and dissolution of the residue in a small amount of methanol (the mixture must be filtrated or centrifuged) we will obtain the extract which is almost free from non polar compounds, e.g., lipids, sterols, *etc.* Before NMR measurements the methanol was evaporated and the sample was dissolved in deuterochloroform.

### 3.3. Samples Analysis

#### 3.3.1. HPLC Analysis

The samples were analyzed using an HP 1050 (Ti-series) HPLC instrument (Hewlett Packard, Palo Alto, CA, USA) on column Luna C18(2) (Phenomenex, Torrance, CA, USA) 3 μm, 150 mm × 2 mm, with water-acetonitrile-*o-*phosphoric acid mobile phase. Mobile phase A: 5% acetonitrile + 0.1% *O*-phosphoric acid; mobile phase B: 80% acetonitrile + 0.1% *o*-phosphoric acid. The gradient was increased from 0% B to 45% B during 55 min and than from 45% B to 80% B during 10 min. Flow rate was 0.250 mL/min and column temperature 25 °C. Injection volume was 5 μL. Diode array detector HP G1315B (DAD, Hewlett-Packard) was used and compounds were detected at 220 and 228 nm. Scanning was carried out in range 190–600 nm. Fluorescence detector HP G1321A (FLD, Hewlett-Packard) was used, with excitation wavelength 315 nm, emission wavelength 395 nm, and scanning of emission was carried out in the range of 300–600 nm. Finally, the method was validated in terms of linearity, limits of detection [17], and repeatability, and it was applied to the analysis of all extracts samples. Detection limit for 2-hydroxy-1,4-naphthoquinone was 0.0018 µg/mL and for 2-methoxy-1,4-naphthoquinone was 0.0016 µg/mL. The content of THNG was calculated according to calibration curve for 2-hydroxy-1,4-naphthoquinone.

#### 3.3.2. LC-MS Analysis

LC-MS was performed using LCQ Accela Fleet (Thermo Fisher Scientific, San Jose, CA, USA) with atmospheric pressure chemical (APCI) and a photodiode array detector. A Phenomenex Luna C18(2) column 3 μm, 150 mm × 2 mm, 18(2) column was used with a water-acetonitrile-formic acid mobile phase. Mobile phase A 5% acetonitrile + 0.1% formic acid; mobile phase B used 80% acetonitrile + 0.1% formic acid. The gradient was increased from 0% B to 45% B during 20 min and from 45% B to 70 B during 5 min. Flow rate was 0.250 mL min^−1^. Injection volume was 5 μL. APCI capillary temperature was 275 °C, APCI vaporizer temperature 450 °C, sheath gas flow 50 L min^−1^, auxiliary gas flow 5 L min^−1^, source voltage 6 kV, source current 5 μA, and capillary voltage −4 V. Methanolic extract of *Impatiens glandulifera* root was diluted 1:2 with methanol and than 1:1 with deionized water before measurement.

#### 3.3.3. NMR measurement

^1^H- and ^13^C-NMR measurements were performed on Varian INOVA 500 MHz spectrometer equipped with standard broadband probe at 25 °C. The structure elucidation was based on 2D experiments (COSY, HSQC, HMBC). The NMR spectra were referenced to the solvent signal: 3.34 and 47.85 ppm for ^1^H and ^13^C, respectively. The obtained shifts are as follows: ^1^H-NMR (CD_3_OD): δ (ppm) 8.35 (d, 1H, H-8), 8.05 (d, 1H, H-5), 7.44 (dt, 1H, H-7), 7.26 (dt, 1H, H-6), 6.54 (s, 1H, H-3), 4.62 (d, 1H, H-1′), 3.88 (dd, 1H, H-6a′), 3.80 (dd, 1H, H-6b′), 3.65 (t, 1H, H-2′), 3.55 (t, 1H, H-4′), 3.48 (t, 1H, H-3′), 3.30 (m, 1H, H-5′). ^13^C-NMR (CD_3_OD): δ (ppm) 151.4 (C_q_-OH, C-4), 146.5 (C_q_-OH, C-2), 131.2 (C_q_-OGlyc, C-1), 129.8 (C_q_, C-9), 126.4 (CH, C-7), 121.9 (2xCH, C-5,6), 121.1 (CH, C-8), 120.5 (C_q_, C-10), 107.1 (CH-ONaph, C-1′), 100.2 (CH, C-3), 77.1 (CH, C-5′), 76.9 (CH, C-3′), 74.4 (CH, C-2′), 69.8 (CH, C-4′), 61.0 (CH_2_, C-6′).

## 4. Conclusions

This work demonstrates the presence and identification of an unstable fluorescent compound which is present in all parts of the *Impatients glandulifera* Royle plants. The possibility of isolation of the compound depend on the drying conditions, storage conditions and on the extraction process. Very clean extract was obtained by the extraction of plant material with ethyl acetate. The structure of compound was assigned as 1,2,4-trihydroxynaphthalene-1-*O*-glucoside according to LC-MS and NMR measurements. The lyophilized roots of *Impatients glandulifera* Royle plant contain up to 826 mg/kg of this compounds when the roots were stored at −18 °C and extracted with methanol, while in the case of the same lyophilized plant material extracted with water as the first solvent, there is no glycoside in the extract. The main compound in this case is 2-hydroxy-1,4-naphthoquinone as a final transformation product of the above newly identified compound. 1,2,4-Trihydroxynaphthalene-1-*O*-glucoside was identified for the first time as a main fluorescent compound in the roots and stems of *Impatients glandulifera* Royle plants. *Impatients glandulifera* Royle contains appreciable amounts of 2-methoxy-1,4-naphthoquinone, the plant is easily accessible and provides a large amount of plant material, it could serve therefore as a source of naphthoquinones. An open question is still the role of THNG in the plants.
